# Higher ambient synaptic glutamate at inhibitory versus excitatory neurons differentially impacts NMDA receptor activity

**DOI:** 10.1038/s41467-018-06512-7

**Published:** 2018-10-01

**Authors:** Lulu Yao, Teddy Grand, Jesse E. Hanson, Pierre Paoletti, Qiang Zhou

**Affiliations:** 1 School of Chemical Biology and Biotechnology, Peking University Shenzhen Graduate School, 518055 Shenzhen, China; 2 Institut de Biologie de l’Ecole Normale Supérieure (IBENS), Ecole Normale Supérieure, CNRS, INSERM, Université PSL, 46 rue d’Ulm, 75005 Paris, France; 3 Department of Neuroscience, Genentech, Inc., South San Francisco, 94080 CA USA

## Abstract

Selective disruption of synaptic drive to inhibitory neurons could contribute to the pathophysiology of various brain disorders. We have previously identified a GluN2A-selective positive allosteric modulator, GNE-8324, that selectively enhances *N*-methyl-d-aspartate receptor (NMDAR)-mediated synaptic responses in inhibitory but not excitatory neurons. Here, we demonstrate that differences in NMDAR subunit composition do not underlie this selective potentiation. Rather, a higher ambient glutamate level in the synaptic cleft of excitatory synapses on inhibitory neurons is a key factor. We show that increasing expression of glutamate transporter 1 (GLT-1) eliminates GNE-8324 potentiation in inhibitory neurons, while decreasing GLT-1 activity enables potentiation in excitatory neurons. Our results reveal an unsuspected difference between excitatory synapses onto different neuronal types, and a more prominent activation of synaptic NMDARs by ambient glutamate in inhibitory than excitatory neurons. This difference has implications for tonic NMDAR activity/signaling and the selective modulation of inhibitory neuron activity to treat brain disorders.

## Abstract (web summary)

Inhibitory interneurons play important roles in brain circuits and in several neuropsychiatric disorders. Here, the authors show that excitatory synapses onto interneurons vs. excitatory neurons differ in their ambient synaptic glutamate level, a finding with important implications for selective pharmacological targeting of inhibitory neuron NMDA receptors.

## Introduction

Inhibitory GABAergic neurons play critical roles in normal brain functions, from precise processing of sensory information to regulation of emotional memories^1–6^. Their malfunction has been implicated in the pathogenesis of many brain diseases, especially psychiatric disorders, including schizophrenia and autism^7–11^. Specifically, reduced function/activity of inhibitory neurons has been documented in various brain disorders, and enhancing their function has thus been proposed and pursued as a potential therapeutic approach. One proposed cause of reduced inhibitory function is *N*-methyl-d-aspartate receptor (NMDAR) hypofunction in inhibitory neurons^9,12–15^. We have recently identified a new series of small-molecule positive allosteric modulators (PAMs) that selectively enhance GluN2A subunit-containing NMDAR currents^16^. Among these PAMs, GNE-8324 is of particular interest since it enhances synaptic NMDAR responses in inhibitory neurons but not excitatory neurons during low-frequency stimulation^16^. Thus, in addition to being useful for boosting inhibitory function in the brain by selectively enhancing inhibitory neuron activity, GNE-8324 may also be used as a tool to explore the differences between inhibitory and excitatory neurons, especially the glutamatergic synapses impinging on them. A deeper understanding of cell-type-specific differences related to the microenvironment surrounding excitatory synapses may lead to new opportunities for therapeutically targeting inhibitory function.

Our previous work indicated a reciprocal allosteric interaction between the GNE-8324 and glutamate binding sites at GluN2A NMDARs such that binding of glutamate enhances binding of GNE-8324 and vice versa^16^. As a consequence, GNE-8324 potentiation is highly dependent on glutamate site occupancy with enhanced GNE-8324 binding to glutamate-bound NMDARs compared to glutamate-free NMDARs. Three potential mechanisms could underlie the selective GNE-8324 potentiation in inhibitory neurons: (1) different NMDAR subunit composition and hence, pharmacology, (2) larger and/or longer glutamate transients during synaptic stimulation, and/or (3) higher ambient glutamate concentration in the synaptic cleft in the absence of synaptic transmission (termed ambient synaptic glutamate), in the inhibitory neurons. These mechanisms could allow greater GNE-8324 association with NMDARs and hence greater potentiation at inputs to inhibitory neurons during synaptic transmission^17^. For example, with a higher ambient synaptic glutamate level and some basal level of NMDAR occupancy by the agonist, GNE-8324 association could occur prior to synaptic stimulation, thus enabling subsequent NMDAR potentiation during synaptic activity (i.e., phasic release of presynaptic glutamate into the synaptic cleft).

In this study, we examined the potential contributions of GluN2 subunits and synaptic glutamate concentration to the selective potentiation of synaptic NMDAR responses by GNE-8324. We found that differences in synaptic NMDAR subunit composition are not responsible for the differential effects of GNE-8324 at synapses onto inhibitory neurons vs. excitatory neurons. We further show that the ambient synaptic glutamate level does indeed play a key role in the selective potentiation of NMDAR excitatory postsynaptic currents (EPSCs) by GNE-8324. In particular, modulating expression/activity of glutamate transporter glutamate transporter 1 (GLT-1) can eliminate GNE-8324 synaptic potentiation in inhibitory neurons or confer synaptic potentiation to excitatory neurons. We performed several different experimental manipulations indicating that ambient synaptic glutamate levels are substantially higher at excitatory synapses onto inhibitory neurons vs. excitatory neurons, thus providing important insight into the diversity of excitatory synapses and cell-type-specific synaptic microenvironments. That the microenvironment at glutamatergic synapses differs between excitatory and inhibitory neurons has broad implications for understanding basic neurophysiology and mechanisms of drug action.

## Results

### GNE-8324 selectively potentiates NMDAR EPSCs in interneurons

**Fig. 1:**
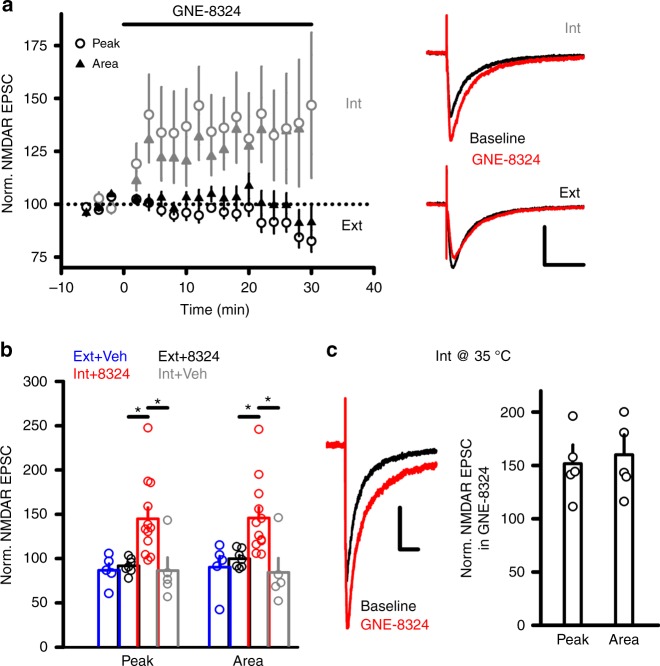
Characterization of potentiation of NMDAR EPSCs by GNE-8324. **a** Effect of GNE-8324 on NMDAR EPSCs in inhibitory neurons (Int, gray) and excitatory neurons (Ext, black). Sample traces of NMDAR EPSCs in inhibitory neurons (Int, upper) and excitatory neurons (Ext, lower) before and after application of GNE-8324. Scale bars, 50 pA/100 ms. **b** Quantification of potentiation shown in **a**. *N* = 5 (Ext + Veh), 7 (Ext + GNE-8324), 12 (Int + GNE-8324), and 5 (Int + Veh). *P < 0.05, one-way ANOVA with Tukey's correction. **c** GNE-8324 potentiation in inhibitory neurons also occurred at 35 °C. Left: Sample traces showing potentiation by GNE-8324. Scale bars, 40 pA/100 ms. Right: Population data. Error bars represent SEM

Our previous study has revealed significant enhancement of NMDAR responses in inhibitory neurons but not excitatory neurons in the hippocampus by GNE-8324^16^. Before studying the mechanism underlying this differential potentiation, we first tested the ability of GNE-8324 to potentiate NMDAR EPSCs in inhibitory neurons under varying conditions. Recordings were made from layer 2/3 of the prefrontal cortex (PFC) at a depth of about 50 to 100 μm in the slices, which did not differ between excitatory and inhibitory neurons. Inhibitory neurons were targeted using GAD67-GFP mice and no distinction between different subtypes of inhibitory neurons was made. NMDAR EPSCs were isolated using blockers of α-amino-3-hydroxy-5-methyl-4-isoxazolepropionic acid (AMPA) receptors ((2,3-dioxo-6-nitro-1,2,3,4-tetrahydrobenzo[*f*]quinoxaline-7-sulfonamide) (NBQX), 10 μM) and γ-amino butyric acid type A (GABA_A_) receptors (picrotoxin, 50 μM) and low Mg^2+^ (0.5 mM). Potentiation of NMDAR EPSCs developed within 5 min after bath application of GNE-8324 (30 μM), with similar increase in both peak and area of NMDAR EPSCs (Fig. 1a, b; *N* = 12; 145.0 ± 12.61% (peak), 145.8 ± 12.03% (area); mean ± SEM). We also found an absence of GNE-8324 potentiation of NMDAR EPSCs in excitatory neurons in the PFC and hence extended our previous observation in the hippocampus (Fig. 1a, b; GNE-8324 + Ext, 91.83 ± 3.27% (peak), 99.88 ± 3.89% (area), *N* = 7; Veh + Ext, 86.75 ± 3.27% (peak), 90.24 ± 12.6% (area), *N* = 5). The rise and decay time of NMDAR EPSCs were not altered by GNE-8324 (Table 1). While these experiments were performed at room temperature, potentiation was also readily observed at 32 °C and 35 °C (Fig. 1c, 35 °C, 150.1 ± 13.80% (peak), 155.8 ± 15.16% (area); *P* (peak) < 0.05, *P* (area) < 0.05, compared to baseline, paired *t* test, *N* = 5; Supplementary Figure 1, 32 °C). Further experiments were performed at room temperature, unless otherwise indicated. The selective potentiation of postsynaptic NMDAR responses in inhibitory neurons by GNE-8324 does not seem to involve presynaptic NMDARs or metabotropic glutamate receptors (mGluRs) as there was no change in paired-pulse ratio (Supplementary Figure 2) after bath application of GNE-8324 and potentiation was also observed in the presence of a broad-spectrum mGluR blocker (*S*)-MCPG ((*S*)*-*α-methyl-4-carboxyphenylglycine) (500 μM) (Supplementary Figure 3).

**Table 1:** Characterization of NMDAR EPSCs in the inhibitory neurons under various conditions

Time (ms)	Baseline- GNE-8324	GNE-8324	Baseline- Pip18	Pip18	Baseline- NAB-14	NAB-14	Baseline- Zinc	Zinc
Rise time (ms) (10–90%)	8.13 ± 0.92	8.23 ± 0.98	6.76 ± 0.32	6.99 ± 0.44	9.89 ± 3.50	10.08 ± 5.10	7.04 ± 1.08	6.27 ± 0.52
*τ*	52.29 ± 7.5	44.81 ± 7.08	44.69 ± 8.479	45.69 ± 8.40	63.94 ± 11.28	64.94 ± 9.68	71.22 ± 10.48	84.25 ± 16.88

*n* (GNE-8324) = 12, *n* (Pip18) = 7, *n* (NAB-14) = 6, *n* (Zinc) = 5

### GluN2A contribution does not explain GNE-8324 selectivity

**Fig. 2:**
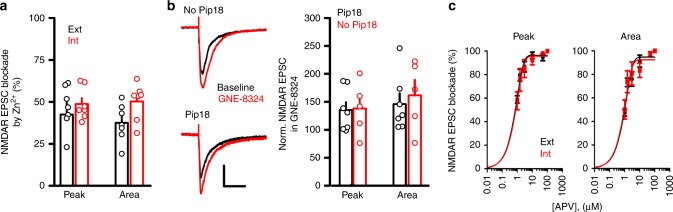
Synaptic GluN2A subunit composition do not underlie inhibitory neuron-selective GNE-8324 potentiation. **a** Similar blockade of NMDAR EPSC by ZnCl_2_ in excitatory (Ext, *N* = 7) and inhibitory (Int, *N* = 7) neurons suggests similar contribution of diheteromeric GluN1/2A NMDARs to NMDAR EPSCs. **b** Similar GNE-8324 potentiation of NMDAR EPSCs in inhibitory neurons in the presence or absence of the GluN2B-selective inhibitor Pip18. Scale bars, 40 pA/100 ms. **c** d-APV inhibits NMDAR EPSCs with similar potency in inhibitory and excitatory neurons. Dose–response curve of NMDAR EPSCs to bath application of d-APV (1, 1.5, 3, 10, 50, and 100 μM) in inhibitory and excitatory neuron. *N* (Ext) = 7, 6, 6, 4, 5, 5; *N* (Int) = 5, 4, 6, 4, 5, 3. Error bars represent SEM

Although the expression levels of GluN2A are high in both excitatory neurons and inhibitory neurons^18,19^, it is possible that synaptic GluN2A-containing NMDARs might be present at a significantly higher density in the inhibitory neurons than in the excitatory neurons, which could account for the selective potentiation by GNE-8324. To test this, we bath applied low concentration of Zn^2+^ (300 nM), which selectively inhibits GluN2A-containing NMDARs^20^. Similar inhibition of synaptic NMDAR EPSCs was seen with Zn^2+^ application in excitatory and inhibitory neurons, suggesting no gross difference in synaptic GluN2A-containing NMDAR content (Fig. 2a, 46.41 ± 4.96% (Peak, Ext), 48.71 ± 3.63% (Peak, Int), 37.51 ± 4.21% (Area, Ext), 50.22 ± 4.15% (Area, Int), *P* (peak) = 0.72, *P* (area) = 0.053, unpaired *t* test, *N* = 7 (Ext), 7 (Int); Table 1). We found that a selective GluN2B antagonist piperidine 18 (1 μM)^21^ did not significantly affect NMDAR EPSCs in the inhibitory neurons (For Pip18, 80.29 ± 4.20% (peak), 80.65 ± 4.06% (area); for Veh, 92.60 ± 5.34% (peak); 91.34 ± 4.15 (area). *P* (peak) = 0.10, *P* (area) = 0.10, *N* = 7 (Pip18), *N* = 5(Veh)), suggesting minimal contribution from diheteromericGluN1/2B  NMDARs. In addition, we found similar potentiation of NMDAR EPSCs by GNE-8324 in the presence or absence of piperidine 18 (Fig. 2b; with Pip18, 135.1 ± 14.43% (peak), 145.9 ± 18.94% (area), *N* = 7; without Pip18, 138.0 ± 21.23% (peak), 161.6 ± 27.49% (area), *N* = 5; *P* (peak) = 0.91, *P* (area) = 0.64, unpaired *t* test; Table 1). We also obtained dose–response curves of NMDAR EPSC inhibition by the competitive antagonist d-(–)-2-amino-5-phosphonopentanoic acid (d-APV) in inhibitory and excitatory neurons to test for differences in d-APV sensitivity of synaptic NMDARs between these cell types. As indicated by the calculated IC_50_ values, we found no significant difference in d-APV sensitivity of NMDAR EPSCs (Fig. 2c; peak, 5.39 μM (Ext) vs. 6.23 μM (Int); area, 9.77 μM (Ext) vs. 8.26 μM (Int)). Together, these experiments suggest that gross differences in ligand sensitivity or the relative amount of GluN2A-containing NMDARs at synapses do not underlie the selective potentiation of excitatory synapses onto inhibitory neurons by GNE-8324.

### Synaptic cleft glutamate is critical to GNE-8324 effects

**Fig. 3:**
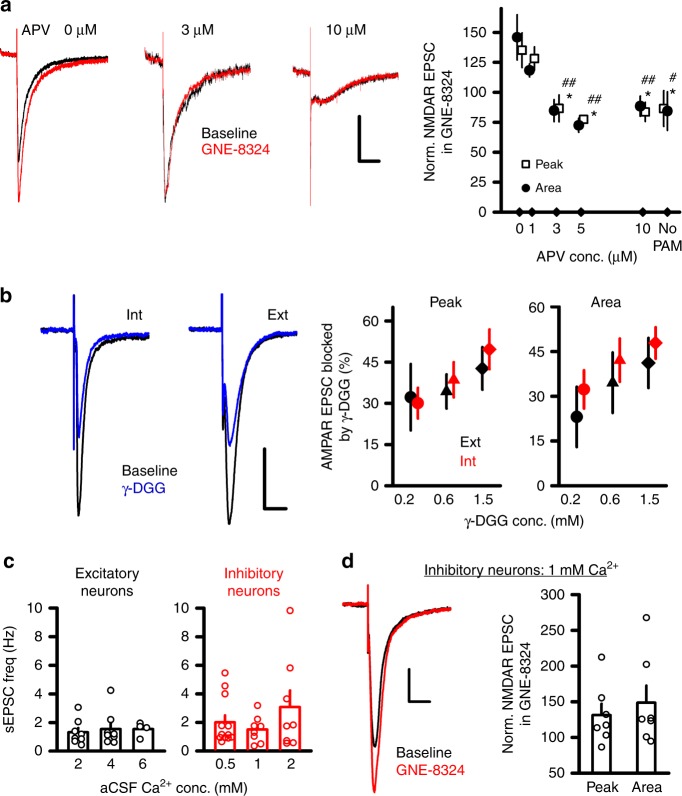
Synaptically released glutamate does not contribute to GNE-8324 potentiation. **a** Submaximal d-APV concentrations eliminates GNE-8324 potentiation in inhibitory neurons. Sample traces of NMDAR EPSCs in inhibitory neurons before and after GNE-8324 application in 0, 3, or 10 μM d-APV. Scale bars, 20 pA/100 ms. Dependence of GNE-8324 potentiation on d-APV concentrations. No PAM (GNE-8324) control (Veh with 0 μM d-APV) shown at the far right. *N* = 7, 5, 9, 3, 7, 5 for 0, 1, 3, 5, 10 μM d-APV and no PAM. All compared to 0 μM d-APV. *P < 0.05; EPSC area. ^#^*P* < 0.05; ^##^*P* < 0.01, one-way ANOVA with Dunnett's correction. EPSC peak amplitude. **b** Similar blockade of AMPAR EPSCs by γ-DGG in excitatory and inhibitory neurons. Sample traces of AMPAR EPSCs in the absence and presence of γ-DGG in inhibitory (Int) and excitatory (Ext) neurons. Scale bars, 50 pA/20 ms. Similar blockade of AMPAR EPSC peak and area by γ-DGG in excitatory and inhibitory neurons across a range of γ-DGG concentrations (0.2, 0.6, and 1.5 mM). 32.21 ± 12.14% (Peak, Ext, 0.2 mM; mean ± SEM), 30.06 ± 5.62% (Peak, Int, 0.2 mM), *P* = 0.88; 34.34 ± 6.22% (Peak, Ext, 0.6 mM), 38.57 ± 6.45% (Peak, Int, 0.6 mM), *P* = 0.66; 42.71 ± 7.78% (Peak, Ext, 1.5 mM), 46.86 ± 6.47% (Peak, Int, 1.5 mM), *P* = 0.56, 23.08 ± 10.13% (Area, Ext, 0.2 mM), 32.28 ± 6.44% (Area, Int, 0.2 mM), *P* = 0.47; 34.55 ± 10.15% (Area, Ext, 0.6 mM), 42.09 ± 7.23% (Area, Int, 0.6 mM), *P* = 0.60; 38.16 ± 9.03% (Area, Ext, 1.5 mM), 49.67 ± 7.24% (Area, Int, 1.5 mM), *P* = 0.39, unpaired *t* test. **c** Spontaneous release of synaptic glutamate does not contribute significantly to GNE-8324 potentiation in inhibitory neurons. Elevating [Ca^2+^]Ext from 2 to 4 or 6 mM did not significantly increase sEPSC frequency in excitatory neurons. 1.32 ± 0.30 Hz (2 mM), 1.44 ± 0.39 Hz (4 mM), 1.54 ± 0.24 Hz (6 mM); *N* = 8, 8, 4. Reducing [Ca^2+^]Ext from 2 to 1 or 0.5 mM reduced sEPSC frequency in inhibitory neurons (2.01 ± 0.48 Hz (0.5 mM), 1.50 ± 0.33 Hz (1 mM), 3.08 ± 1.16 Hz (2 mM); *N* = 12, 8, 8). **d** Significant GNE-8324 potentiation in inhibitory neurons at 1 mM [Ca^2+^]Ext. Scale bars, 50 pA/50 ms. Error bars represent SEM

As presented in the Introduction, one possible mechanism underlying the differential potentiation by GNE-8324 in inhibitory neurons is a higher glutamate level in the synaptic cleft either prior to or during synaptic transmission. To test this, we used the high-affinity competitive antagonist d-APV to reduce the effective glutamate concentration at synaptic NMDARs by constitutive displacement of glutamate agonist molecules from synaptic NMDARs (i.e., reduce agonist occupancy), which could reduce GNE-8324 occupancy at the time of synaptic stimulation. We found that d-APV eliminated GNE-8324 potentiation of NMDAR EPSCs in inhibitory neurons when the d-APV concentration was 3 μM or higher (Fig. 3a; in 1 μM d-APV, 128.2 ± 9.72% (peak), 118.3 ± 4.50% (area); in 10 μM d-APV, 83.6 ± 8.02% (peak), 88.39 ± 8.35% (area); *P* (peak) < 0.05, *P* (area) < 0.01, one-way analysis of variance (ANOVA) with Dunnett's correction; *N* = 5 (1 μM) and 7 (10 μM)), suggesting that higher glutamate concentration in the synaptic cleft in inhibitory neurons may underlie the selective GNE-8324 potentiation. In this experiment, d-APV was present throughout the entire experiment and hence potentiation in inhibitory neurons was reduced when the effective glutamate level was lowered both before and during synaptic transmission. Thus, it is possible that either higher ambient synaptic glutamate levels or higher glutamate transients during synaptic transmission are important for allowing GNE-8324 potentiation in inhibitory neurons.

### Glutamate concentration during transmission is not critical

Next, we tested whether the glutamate concentration during synaptic transmission is higher at glutamatergic synapses on inhibitory neurons compared to excitatory neurons. Synaptic glutamate concentration cannot be measured directly, but a qualitative comparison can be made from the effects of a low-affinity competitive AMPAR antagonist γ-d-glutamylglycine (γ-DGG). Blockade of AMPAR EPSCs by γ-DGG depends on the synaptic glutamate concentration in that higher glutamate concentrations result in smaller blockade^22^. Bath application of γ-DGG did not reveal a significant difference in the degrees of reduction in AMPAR EPSCs between inhibitory and excitatory neurons, either in the area or peak (Fig. 3b; *N* = 5, 5 (0.2 mM); 6, 4 (0.6 mM); 8, 5 (1.5 mM), for excitatory and inhibitory neuron, respectively) across a range of concentrations tested (0.2, 0.6, and 1.5 mM). In the above experiments, 10 μM d-APV was used to block NMDARs. In additional experiments, we used higher concentration of d-APV (100 μM) to fully block NMDARs to exclude the possibility that γ-DGG could affect NMDARs, and we still found no difference between blockade of AMPAR EPSCs by γ-DGG (Supplementary Figure 4). This suggests no significant difference in the glutamate level during synaptic transmission between excitatory and inhibitory neurons, which is consistent with the similar profiles of NMDAR EPSC inhibition by d-APV (Fig. 2c).

In addition to evoked synaptic transmission, ongoing spontaneous release of glutamate from synaptic vesicles may contribute to the glutamate concentrations in the synaptic cleft in the absence of evoked synaptic transmission. We first recorded spontaneous EPSCs (sEPSCs) under the same condition as in the experiments in Fig. 1, and found that sEPSCs showed a trend towards higher frequency in the inhibitory neurons than in the excitatory neurons (Fig. 3c; 1.32 ± 0.30 Hz (2 mM, Ext) vs. 3.08 ± 1.16 Hz (2 mM, Int); *P* = 0.16, unpaired *t* test). This potential difference could be caused by higher synapse density in the inhibitory neurons and/or a higher probability of glutamate release from the presynaptic terminals connecting to postsynaptic inhibitory neurons. In either case, more spontaneous synaptic transmission could lead to higher ambient synaptic glutamate level due to potential spillover between neighboring synapses^23^. To address whether sEPSC could contribute to selective GNE-8324 potentiation, we altered presynaptic release probability by changing Ca^2+^/Mg^2+^ ratio in the recording artificial colony-stimulating factor (aCSF). We found that elevating [Ca^2+^]Ext from 2 to 4 mM or 6 mM while keeping [Mg^2+^]Ext at 0.5 mM did not significantly increase sEPSC frequency in the excitatory neurons (Fig. 3c), but reducing [Ca^2+^]Ext to 1 mM reduced sEPSC frequency in the inhibitory neurons to a level similar to that in the excitatory neurons with [Ca^2+^]Ext at 2 mM (Fig. 3c; 1.50 ± 0.33 Hz (1 mM, Int), 1.32 ± 0.30 Hz (2 mM, Ext)). Therefore, we tested GNE-8324 on NMDAR EPSCs in the inhibitory neurons using 1 mM [Ca^2+^]Ext, and still found significant GNE-8324 potentiation (Fig. 3d; 131.4 ± 15.77% (peak), 148.7 ± 23.83 (area); *P* (peak) = 0.63, *P* (area) = 0.93, compared to GNE-8324 in 2 mM [Ca^2+^]Ext, unpaired *t* test; N = 7). Taken together, the above results indicated that synaptically released glutamate, via either stimulated release or spontaneous release, does not contribute significantly to the preferential GNE-8324 potentiation of NMDAR EPSCs in inhibitory neurons.

### Lower ambient synaptic glutamate abolishes GNE-8324 effects

**Fig. 4:**
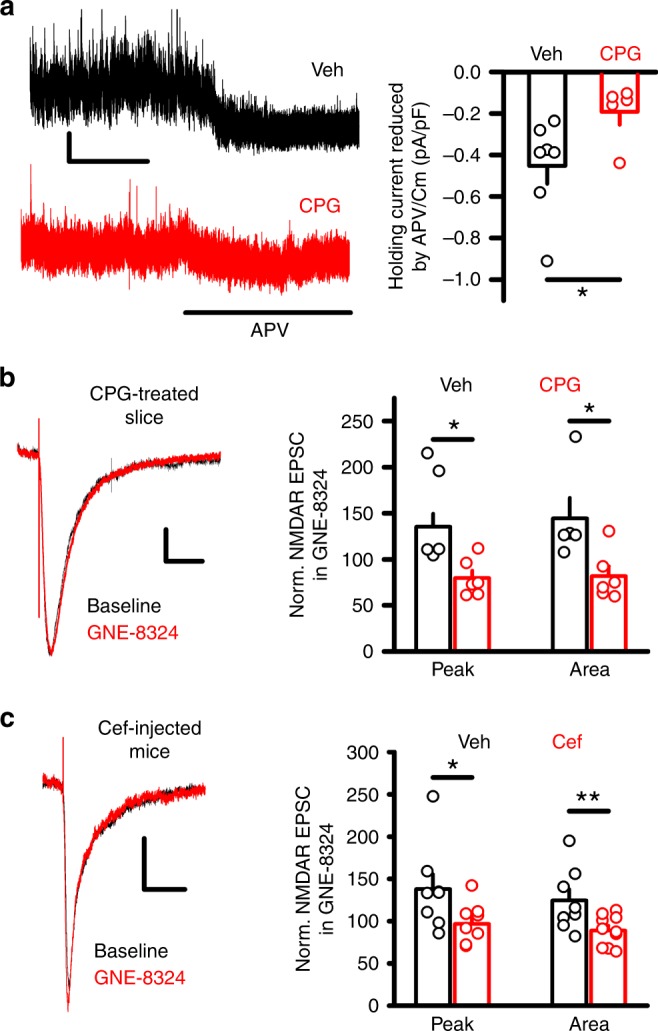
Ambient glutamate and glutamate transporters play critical roles in GNE-8324 potentiation of NMDAR EPSCs in inhibitory neurons. **a** Reduced NMDAR responses to ambient glutamate after CPG treatment. Sample traces showing reduced d-APV-induced holding current changes in inhibitory neurons treated with CPG. Scale bars, 10 pA/500 ms. d-APV-induced decrease in holding current was significantly smaller in CPG-treated neurons than in vehicle-treated neurons. *P < 0.05, unpaired *t* test. **b** CPG treatment eliminated GNE-8324 potentiation in the inhibitory neurons. Sample traces of NMDAR EPSCs before and after GNE-8324 application in the inhibitory neurons incubated with CPG. Scale bars, 20 pA/100 ms. Compared to the robust potentiation in vehicle-treated neurons, CPG-treated neurons showed no significant GNE-8324 potentiation of NMDAR EPSCs. *P < 0.05, unpaired *t* test. **c** GNE-8324 potentiation was eliminated in the inhibitory neurons from mice injected with ceftriaxone (Cef). Sample traces of NMDAR EPSCs before and after GNE-8324 application from an inhibitory neuron in Cef-injected mouse. Scale bars, 10 pA/100 ms. Effect of GNE-8324 in inhibitory neurons from Cef-injected and Veh-injected mice. **P < 0.01, unpaired *t* test. Error bars represent SEM

The above results suggest that the differential potentiation of GNE-8324 in inhibitory neurons is unlikely caused by difference in the GluN2A NMDAR subunit composition or in glutamate concentrations in the synaptic cleft during either evoked or spontaneous release. A remaining possibility is that differences in ambient synaptic glutamate levels might explain the selective potentiation by GNE-8324. Previous work has shown that ambient (extracellular) glutamate levels can be altered by modulating the activity of cysteine-glutamate exchanger, using compounds such as 4-(*s*)-carboxyphenylglycing (CPG)^24^. To quantify the effect of CPG (50 μM) on ambient glutamate levels, we measured changes in holding current in response to application of d-APV at +40 mV, which has been used widely as a measure of ambient glutamate-induced NMDAR responses^25–28^. d-APV-induced changes in holding current were significantly smaller in CPG-treated inhibitory neurons compared to vehicle-treated neurons (Fig. 4a; *P* < 0.05, unpaired *t* test, *N* = 7 (vehicle), 5 (CPG)), consistent with reduced ambient glutamate level by CPG treatment. Under this condition of reduced ambient glutamate, GNE-8324 no longer potentiated NMDAR EPSCs in the inhibitory neurons from CPG-treated slices, in contrast to robust potentiation in the vehicle-treated slices (Fig. 4b; Veh, 147.4 ± 23.94% (amp), 144.2 ± 22.50% (area); CPG, 79.59 ± 8.23% (amp), 81.58 ± 10.81% (area); *P* (peak) < 0.05, *P* (area) < 0.05, unpaired *t* test; *N* = 6 (CPG) and 5 (Veh)). The above results indicate that reducing the ambient glutamate level eliminates GNE-8324 potentiation in inhibitory neurons, suggesting that higher ambient glutamate level at synaptic NMDARs in inhibitory neurons is a key factor for the selective GNE-8324 potentiation.

Glutamate transporters, especially those on the astrocytes (such as GLT-1), are responsible for clearing glutamate from the synaptic cleft after synaptic transmission^29–31^, and these transporters could also be critically involved in regulating the accessibility of ambient glutamate to the synaptic cleft and synaptic NMDARs^32,33^. GLT-1 (EAAT2) is the most abundantly expressed glutamate transporter among the five glutamate transporters (EAAT1-5), and accounts for approximately 95% of glutamate uptake^34–38^. The density of GLT-1 surrounding glutamatergic synapse on inhibitory neurons is lower than the density surrounding synapse on Purkinje cells in the cerebellum^39^, and it is possible that similar differences in GLT-1 density could occur at synapses onto different cell types in the forebrain. We hypothesized that a lower density of GLT-1 on astrocytes at glutamatergic synapses on inhibitory neurons results in a higher ambient synaptic glutamate level in inhibitory neurons compared to excitatory neurons. If this is correct, enhancing GLT-1 levels should reduce ambient synaptic glutamate levels and eliminate GNE-8324 potentiation. To test this, we injected GAD67-GFP mice with ceftriaxone (Cef) (200 mg/kg/day, intraperitoneal (i.p.)), a β-lactam antibiotic that effectively increases the expression of GLT-1^40–42^. We confirmed that Cef injection for 5 consecutive days led to significantly elevated GLT-1 expression using western blot (Supplementary Figure 5). In recordings using mice that received Cef injections, we found that GNE-8324 lost its potentiation of NMDAR EPSCs in inhibitory neurons, while significant potentiation was observed in slices from vehicle-injected mice (Fig. 4c; Veh, 137.8 ± 17.71% (peak), 124.5 ± 13.13% (area); Cef, 96.78 ± 7.48% (peak), 88.98 ± 5.647% (area); *P* (peak) < 0.05, *P* (area) < 0.01, unpaired *t* test; *N* = 10 (Cef), 8 (Veh)). This result supports the hypothesis that strong glutamate uptake through high GLT-1 levels can prevent GNE-8324 potentiation.

### Raising ambient synaptic glutamate enables GNE-8324 effects

**Fig. 5:**
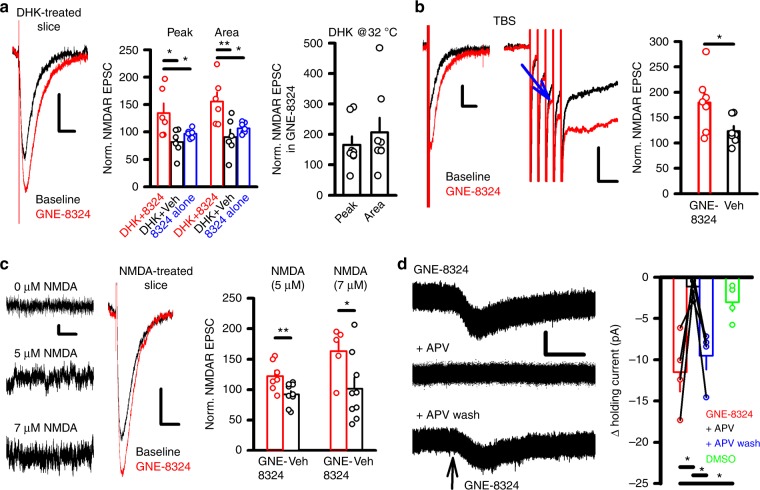
Increasing ambient synaptic glutamate level in the excitatory neurons enables GNE-8324 potentiation of NMDAR EPSCs. **a** Bath application of the GLT-1 inhibitor DHK revealed significant potentiation of NMDAR EPSCs in the excitatory neurons. Sample traces of NMDAR EPSCs before and after GNE-8324 in the presence of DHK. Scale bars, 10 pA/100 ms. GNE-8324 potentiation was only observed in excitatory neurons following DHK treatment. *P (peak) < 0.05; **P (area) < 0.01, one-way ANOVA with Dunnett's correction, both compared to vehicle. Significant GNE-8324 potentiation was observed at 32 °C in the presence of DHK. **b** TBS in the excitatory neurons revealed significant GNE-8324 potentiation. (Left) Sample traces showing prominent GNE-8324 potentiation. (Right) Expanding the portion of EPSC traces around the TBS portion revealed increasingly larger baseline responses with successive TBS pulses (indicated by the arrow). Scale bars, 10 pA/100 ms (left); 20 pA/10 ms (right). TBS-induced NMDAR EPSC areas were significantly enhanced by GNE-8324, compared to vehicle. *P < 0.05, unpaired *t* test. **c** Bath-applied NMDA enabled GNE-8324 potentiation in the excitatory neurons. Sample traces of holding current after bath application of 0, 5, or 7 μM NMDA. Scale bars, 5 pA/100 ms. Sample traces showing significant GNE-8324 potentiation in the excitatory neurons in the presence of 7 μM NMDA. Scale bars, 10 pA/100 ms. Quantification of GNE-8324 potentiation in the presence of NMDA. For 5 μM NMDA, GEN-8324, 122.2 ± 9.05% (area); Veh, 92.33 ± 5.39 (area); **P (area) < 0.01; *N* = 10 (GNE-8324) and *N*  = 7 (Veh). For 7 μM NMDA, GNE-8324, 163.3 ± 18.53% (area), 101.6 ± 17.94% (area); *P (area) < 0.05; *N* = 9 (GNE-8324) and *N*  = 5 (Veh), unpaired *t* test. **d** GNE-8324 potentiated NMDAR responses in excitatory neurons in the absence of synaptic stimulation. Sample traces. Puffing of GNE-8324 (3 mM) is indicated by the arrow. Scale bars, 10 pA/1 s. Quantification of responses to puffing of GNE-8324. *N* = 4 (GNE-8324), 4 (DMSO), 4 (GNE-8324 in APV), and 4 (GNE-8324 after APV washout). **P < 0.01, paired *t* test. The same neurons are connected by lines. Error bars represent SEM

If a higher ambient glutamate level in the synaptic cleft is a key factor in GNE-8324 potentiation in inhibitory neurons, it follows that elevating ambient synaptic glutamate in the excitatory neurons should enable GNE-8324 potentiation. Specifically, if GLT-1 is critically involved in regulating the glutamate level, we expect that blocking GLT-1 will enable GNE-8324 potentiation in the excitatory neurons. Bath application of dihydrokainate (DHK) (300 μM), a selective GLT-1 inhibitor^43–46^, significantly increased NMDAR EPSCs (Supplementary Figure 6), consistent with previous reports^47,48^. While GNE-8324 alone had no effect in excitatory neurons in the absence of DHK, it readily potentiated NMDAR EPSCs in the presence of DHK (Fig. 5a; DHK + GNE-8324: 134.7 ± 17.61% (peak), 155.5 ± 17.09% (area), *N* = 6; DHK + Veh: 81.6 ± 9.58% (peak), 90.5 ± 13.69% (area), *N* = 6; *P* (peak) < 0.05; *P* (area) < 0.01, one-way ANOVA with Dunnett's correction, DHK + GNE-8324 vs. DHK + Veh; GNE-8324 alone: 96.9 ± 2.85% (peak), 106.8 ± 3.66% (area), *N* = 7; P (peak) < 0.05; P (area) < 0.05, one-way ANOVA with Dunnett 'scorrection,DHK + GNE-8324 vs. GNE-8324). Again, GNE-8324 potentiation was about the same magnitude on both the peak and area of NMDAR EPSCs, and was also observed at 32 °C (Fig. 5a; 165.5 ± 27.88% (peak), 207.4 ± 46.60% (area), *N* = 8; *P* (peak) = 0.41, P (area) = 0.37, unpaired *t* test, compared to DHK + 8324 at room temperature). To exclude the possibility that the observed DHK effect was due to effect other than blocking glutamate transporters, we also tested whether a different (and more broad-spectrum) glutamate transporter blocker, dl-threo-β-benzyloxyaspartate (dl-TBOA) (10 μM)^49^, could also enable GNE-8324 potentiation in excitatory neurons. In these experiments using dl-TBOA, we also found significant potentiation of NMDAR EPSCs in excitatory neurons by GNE-8324 (Supplementary Figure 7).

Under physiological conditions, brief high-frequency synaptic activity (such as *θ*-burst stimulation, TBS) can significantly reduce glutamate transporter capacity, thus resulting in glutamate build-up in both the synaptic cleft and extracellular space^50^. We delivered TBS to excitatory neurons and saw significant GNE-8324 potentiation (Fig. 5b, GNE-8324 vs. Veh; TBS + GNE-8324: 157.8 ± 18.96% (peak), 179.4 ± 21.43% (area), *P* (area) < 0.05, unpaired *t* test; *N* = 7, 7 for TBS and Veh). Close inspection of NMDAR EPSC traces during TBS revealed that in the presence of GNE-8324, potentiation of the NMDAR EPSCs only emerged during later pulses in the TBS (Fig. 5b), consistent with increased ambient synaptic glutamate levels as glutamate transporters are saturated.

Next, we asked whether exogenously applied NMDA can also enable GNE-8324 potentiation. The rationale being that sufficiently high concentration of exogenous NMDA should lead to an increase in ambient agonist level in the synaptic cleft, since NMDA is not taken up by glutamate transporters^37,51^. Progressively elevating the exogenous NMDA concentration resulted in an increase in baseline noise and holding current (Fig. 5c; Supplementary Figure 8)^52,53^, indicating elevated activation of NMDARs due to increased agonist level. We found a small but significant GNE-8324 potentiation with 5 μM NMDA and greater potentiation with 7 μM NMDA in excitatory neurons (Fig. 5c; *P* < 0.01 (5 μM NMDA vs. Veh) and *P* < 0.05 (7 μM NMDA vs. Veh), unpaired *t* test), consistent with elevated agonist binding to NMDARs allowing sufficient binding of GNE-8324.

Together, these results suggest ambient synaptic glutamate allows GNE-8324 potentiation of synapses on inhibitory neurons, but tight regulation of glutamate specifically at synapses onto excitatory neurons prevents potentiation. This suggests that extrasynaptic NMDARs on excitatory neurons, which could be exposed to higher ambient glutamate levels than synaptic NMDARs, might be able to be potentiated by GNE-8324. Therefore, we tested whether GNE-8324 could potentiate extrasynaptic NMDARs in excitatory neurons by puffing GNE-8324 (3 mM) onto excitatory neurons while they were held at −60 mV in 0.5 mM Mg^2+^ (with AMPARs/GABA_A_Rs blocked). We observed a significant inward current following GNE-8324 puffing which was blocked by bath application of d-APV and reemerged after washout of d-APV in the same neurons (Fig. 5d; *P* < 0.05, GNE-8324 vs. GNE-8324 + APV; *P* < 0.05, GNE-8324 vs. dimethyl sulfoxide (DMSO), paired *t* test), consistent with potentiation of extrasynaptic NMDAR activated by ambient glutamate.

Overall, the above evidence suggests that there are significant differences in the ambient synaptic glutamate level between inhibitory and excitatory neurons, and this difference is likely regulated by a difference in the level/activity of glutamate transporters (such as GLT-1), which results in the differential potentiation by GNE-8324.

### No role of triheteromeric GluN2C/D-containing NMDARs

The above results are consistent with GNE-8324 potentiation being possible in inhibitory neurons due to higher ambient glutamate levels within the synaptic microenvironment. However, another formal possibility consistent with these results is that the glutamate levels are unchanged, but (at least a fraction of) synaptic NMDARs in inhibitory neurons are more sensitive to glutamate and can therefore by potentiated. In particular, expression of GluN2C/GluN2D-containing NMDARs is higher in inhibitory neurons^54–57^ and NMDARs composed of GluN2C or GluN2D subunits have higher glutamate sensitivity^15^. This could theoretically allow greater GNE-8324 potentiation of triheteromeric GluN1/2A/2C and/or GluN1/2A/2D NMDARs in inhibitory neurons.

**Fig. 6:**
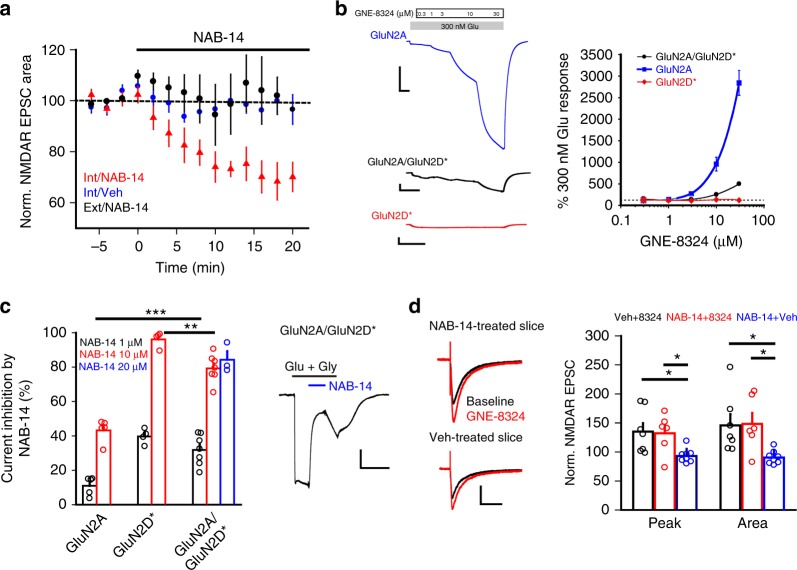
GluN2C/D-containing NMDARs contribute to NMDAR EPSCs but not GNE-8324 potentiation in inhibitory neurons. **a** NAB-14 reduces NMDAR EPSC areas in inhibitory but not excitatory neurons. **b** Sensitivity to GNE-8324. Left: Current trace examples of GNE-8324 dose–response recordings from oocytes expressing either diheteromeric GluN1/GluN2A or GluN1/GluN2D* NMDARs, or triheteromeric GluN1/GluN2A/GluN2D* NMDARs. Scale bars, 200 nA/50 s (top), 20 nA/50 s (middle), and 10 nA/50 s (bottom). Right: GNE-8324 dose–response curves for GluN1/GluN2A, GluN1/GluN2D*, or GluN1/GluN2A/GluN2D* NMDARs. *N* = 5–7 oocytes/concentration. **c** Sensitivity to NAB-14. Effect of 1 and 10 µM NAB-14 on diheteromeric GluN1/GluN2A NMDARs, diheteromeric GluN1/GluN2D* NMDARs, and triheteromeric GluN1/GluN2A/GluN2D* NMDARs. Later, 20 µM NAB-14 was also tested. Right: Illustrative current trace showing inhibition of triheteromeric GluN1/GluN2A/GluN2D* NMDAR activity by 20 µM NAB-14. Scale bars, 20 nA/40 s. *N* = 3–7 oocytes/concentration. **P < 0.01, ***P < 0.001, one-way ANOVA with Tukey's correction. **d** NAB-14 does not affect the level of GNE-8324 potentiation on NMDAR EPSCs in inhibitory neurons. Left: Sample traces showing similar potentiation of NMDAR EPSCs by GNE-8324 in NAB-14-treated slice and Veh-treated slice. Scale bars, 50 pA/100 ms. *N* = 7, 6, 8, for Veh + 8324, NAB-14 + 8324, and NAB-14 + Veh, respectively. *P < 0.05, one-way ANOVA with Dunnett's correction. Right: Population data. Error bars represent SEM

To examine whether GluN2C/2D-containing NMDARs may contribute to GNE-8324 potentiation, we used NAB-14 (*N*-aryl benzamide), a selective antagonist of GluN2C/2D-containing NMDARs (diheteromeric GluN1/2C and GluN1/Glu2D NMDARs, as well as triheteromeric GluN1/2A/2C NMDARs)^57^. A significant reduction in NMDAR EPSCs was seen after 20 μM NAB-14 application in inhibitory neurons, but not in excitatory neurons (Fig. 6a; Supplementary Figure 9; 71.70 ± 3.54% (peak, Int/NAB-14), 95.20 ± 4.25% (peak, Int/Veh); 71.92 ± 4.31% (area, Int/NAB-14), 101.0 ± 3.25% (area, Int/Veh); 93.95 ± 7.91% (peak, Ext/NAB-14), 103.7 ± 10.92% (area, Ext/NAB-14); *P* (peak) < 0.05, *P* (area) < 0.05, Int/Veh vs. Int/NAB-14; *P* (peak) < 0.05, *P* (area) < 0.05, Ext/NAB-14 vs. Int/NAB-14, all one-way ANOVA with Dunnett's correction), demonstrating a preferential contribution of GluN2C/2D-containing NMDARs to NMDAR EPSCs in inhibitory neurons, consistent with previous findings^54–57^. Next, we examined whether triheteromeric GluN1/2A/2D NMDARs have high glutamate sensitivity (see Methods). Using a strategy previously described to isolate triheteromeric NMDARs in heterologous systems^58^, we selectively examined the properties of recombinant GluN1/2A/2D NMDARs. We found that glutamate sensitivity of triheteromeric GluN1/2A/2D NMDARs is higher than that of diheteromeric GluN1/2A NMDARs (but lower than that of diheteromeric GluN1/2D NMDARs) (Supplementary Figure 10). This raises the possibility that inhibitory neurons could contain a population of triheteromeric NMDARs with increased glutamate sensitivity. However, we found that potentiation of triheteromeric GluN1/2A/2D NMDARs by GNE-8324 is much smaller than that of diheteromeric GluN1/2A NMDARs (Fig. 6b), arguing against a strong involvement of triheteromeric GluN1/2A/2D NMDARs in GNE-8324 potentiation of NMDAR EPSCs in inhibitory neurons.

Although NAB-14 has been shown to inhibit triheteromeric GluN1/2A/2C NMDARs^57^, it is unknown whether it can also effectively inhibit triheteromeric GluN1/2A/2D NMDARs, a NMDAR subtype that likely constitute a significant portion of synaptic NMDARs on inhibitory neurons^54–57^. We thus assessed the NAB-14 sensitivity of triheteromeric GluN1/2A/2D NMDARs and found that NAB-14 applied at 10 µM or above produced strong inhibition of GluN1/2A/2D NMDARs (Fig. 6c; 84 ± 0.05% inhibition by 20 µM NAB-14, *N* = 3; *P* < 0.01 (GluN1/2D vs. GluN1/2 A/2D), *P* < 0.0001 (GluN1/N2A vs. GluN1/2 A/2D), unpaired *t* test). Given this result, we next tested if GNE-8324 potentiation of synaptic NMDARs in inhibitory neurons can still be observed when GluN2C/D-containing NMDARs (including GluN1/2A/2D triheteromers) are pharmacologically silenced by NAB-14. We found no significant difference in the potentiation by GNE-8324 of NMDAR EPSCs in the presence or absence of 20 μM NAB-14 (Fig. 6d; 132.2 ± 13.95% (peak, NAB-14), 135.1 ± 14.43% (peak, no NAB-14), *P* = 0.89; 148.4 ± 18.87% (area, NAB-14), 145.9 ± 18.94% (area, no NAB-14), *P* = 0.93, unpaired *t* test). GluN2A-containing triheteromeric NMDARs (GluN1/2A/2C and/or GluN1/2A/2D receptors) are thus unlikely to mediate GNE-8324 potentiation. Overall, these results strongly suggest that GluN2 subunit composition and associated changes in glutamate sensitivity do not contribute significantly to the selective potentiation of GNE-8324 in inhibitory neurons.

### Tonic activation of synaptic NMDARs on inhibitory neurons

**Fig. 7:**
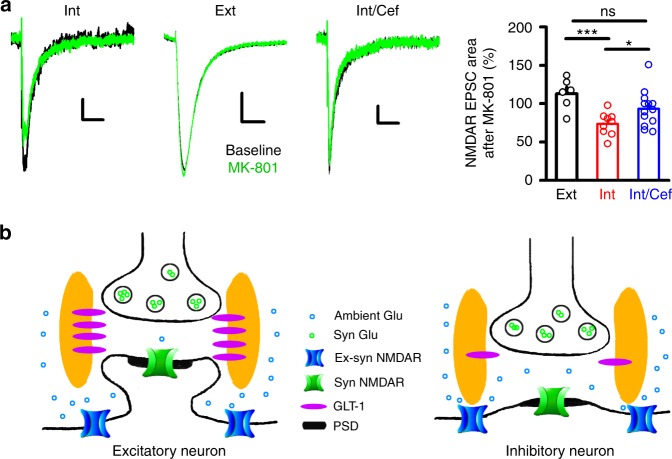
Differential impact of ambient glutamate on synaptic NMDAR responses in excitatory and inhibitory neurons. **a** Ambient glutamate activation of synaptic NMDARs was assayed by measuring the use-dependent blockade by MK-801 in the absence of synaptic stimulation (see text). Synaptic NMDAR EPSCs were reduced by MK-801 application in the absence of synaptic stimulation in inhibitory neurons but not excitatory neurons and this reduction was ameliorated in inhibitory neurons from Cef-treated mice. Sample traces of NMDAR EPSCs before and after MK-801 application in an inhibitory neuron (left), an excitatory neuron (middle), or an inhibitory neuron from a Cef-treated mouse (right). Scale bars, 10 pA/100 ms (left); 30 pA/100 ms (middle); 10 pA/100 ms (right). No significant change in NMDAR EPSCs area was observed after MK-801 in excitatory neurons (*N* = 6), while a significant reduction was observed in inhibitory neurons (*N* = 9; ***P < 0.001, 113.1 ± 8.25% (Ext), 73.67 ± 4.97% (Int), unpaired *t* test, compared to excitatory neurons). Inhibitory neurons in Cef-treated mice showed a significantly reduced effect of MK-801 application (*N* = 13; 73.67 ± 4.97% (Int), 93.44 ± 6.52% (Ext). *P < 0.05, unpaired *t* test, compared to inhibitory neurons without Cef injection). **b** A model depicting the differential resting glutamate levels in the synaptic cleft in excitatory (left) and inhibitory (right) neurons, likely caused by difference in the density/activity of glutamate transporters (such as GLT-1s). Error bars represent SEM. ns not significant

Together, the above experiments suggest that higher ambient synaptic glutamate levels allow GNE-8324 potentiation of synaptic NMDARs, which suggests that ambient glutamate is higher at synaptic locations on inhibitory neurons. Does this higher ambient synaptic glutamate affect the function of synaptic NMDARs? To test this, we used the irreversible, activity-dependent NMDAR antagonist MK-801 to assess the level of ambient synaptic glutamate-induced activation of synaptic NMDARs, and whether this level differs between excitatory and inhibitory neurons. We first recorded NMDAR EPSCs (−60 mV, 0.5 mM Mg^2+^) to establish a baseline response, and paused stimulation while voltage-clamping the postsynaptic neurons at +40 mV in the presence of MK-801 (20 μM) to block synaptic NMDARs activated by ambient glutamate^25,59^. After 5 min, the holding potential was switched back to −60 mV with synaptic stimulation resumed. The reduction in NMDAR EPSCs at this point was used as a functional readout of ambient glutamate activation of synaptic NMDARs. As MK-801 is slow to wash out of slices, only the first three NMDAR EPSCs after resuming stimulation were measured before stimulation-dependent blockade could accumulate. This procedure led to a significant reduction in the peak and area of NMDAR EPSCs in the inhibitory neurons, but virtually no change in the excitatory neurons (Fig. 7a, *P* < 0.001, unpaired *t* test. Ext vs. Int; *N* = 6 (Ext), 9 (Int)). This suggests significantly greater activation of synaptic NMDARs by ambient glutamate in inhibitory neurons. While this interpretation requires several assumptions (equal ability of MK-801 to block receptors with different subunit compositions, little or no recovery during the time frame of the experiments, etc.), additional data support a role for ambient synaptic glutamate. In particular, we observed that the reduction in NMDAR EPSCs was much less pronounced in inhibitory neurons from Cef-treated mice (Fig. 7a). This is consistent with Cef injection, which increases GLT-1 function, reducing the impact of ambient glutamate on synaptic NMDARs in inhibitory neurons. Thus, these experiments using MK-801 provide an independent line of evidence from the GNE-8324 experiments, which also indicates higher ambient synaptic glutamate levels at synapses onto inhibitory neurons vs. excitatory neurons.

## Discussion

In this study, we provide substantial evidence that the preferential enhancement of NMDAR EPSCs in inhibitory neurons vs. excitatory neurons by the GluN2A PAM GNE-8324 is mediated by a higher ambient synaptic glutamate level, and that this higher glutamate level may result from lower glutamate transporter GLT-1 expression/activity. These findings deepen our understanding of the fundamental differences between excitatory and inhibitory neurons, and also have implications for treating brain diseases involving altered inhibitory neuron function.

GNE-8324 selectively potentiates GluN2A subunit-containing NMDARs^16^. We found similar reduction of NMDAR EPSCs in inhibitory and excitatory neurons by a concentration of Zn^2+^ that is selective for GluN2A-containing NMDARs, suggesting that differences in synaptic GluN2A NMDAR proportion are not the main source of GNE-8324 selectivity for inhibitory neurons. GNE-8324 potency is enhanced when NMDARs are bound by glutamate, and as discussed below, potentiation of synaptic NMDARs may require their activation by ambient glutamate. We find that synaptic NMDARs on PFC inhibitory neurons include GluN2C/D-containing NMDARs (which exhibit greater sensitivity to glutamate). However, while we find that triheteromeric GluN1/2A/2D NMDARs also have an increased sensitivity to glutamate, GNE-8324 potentiation of these receptors is much smaller than on diheteromeric GluN1/2A NMDARs. Critically, GNE-8324 potentiation of synaptic NMDARs on inhibitory neurons persists even when these higher glutamate sensitivity GluN2C/2D-containing NMDARs are blocked by NAB-14. Thus, the presence of GluN2C/2D NMDARs does not play a significant role in the selective GNE-8324 potentiation seen in inhibitory neurons.

A high glutamate level in the synaptic cleft appears critical for GNE-8324 potentiation in inhibitory neurons, as indicated by the absence of GNE-8324 potentiation when the NMDAR antagonist d-APV was used to reduce the effective glutamate concentration (Fig. 3a). We have examined three potential sources of elevated synaptic glutamate in inhibitory neurons: (1) different glutamate profiles during synaptic transmission, (2) potentially greater spontaneous release of glutamate, and (3) reduced glutamate transporter density/function resulting in higher ambient synaptic glutamate levels. We excluded the first possibility based on the similar blockade of γ-DGG on synaptic AMPAR responses, and the second possibility based on robust GNE-8324 potentiation in inhibitory neurons even when sEPSC frequency was reduced to the level seen in excitatory neurons.

We addressed the possibility of higher ambient synaptic glutamate level in inhibitory neurons using multiple approaches. There are two potential major sources of this ambient synaptic glutamate: diffusion of non-synaptic glutamate from the extrasynaptic space (extracellular glutamate) and residual synaptic glutamate from previous synaptic activity^60^. Both are likely subject to regulation by glutamate transporters (especially GLT-1). GNE-8324 lost its potentiation in the inhibitory neurons when the extracellular glutamate concentration was reduced by CPG, or the density of GLT-1s was elevated via injection of Cef. In the converse experiments, we found that GNE-8324 induced robust potentiation in excitatory neurons with: (1) reduction of GLT-1 activity by its selective blocker DHK, (2) reduction of glutamate transporter activity by the broad-spectrum glutamate transporter inhibitor dl-TBOA, (3) high-frequency synaptic stimulation (TBS), and (4) exogenous application of NMDA. All four acute manipulations, which elevate the ambient synaptic glutamate/agonist level, should have minimal impact on the composition and properties of NMDARs. Although each approach has its limitation or caveat, the fact that they all revealed significant GNE-8324 potentiation strongly suggests that it is the glutamate level rather than the intrinsic properties of NMDARs that differ between excitatory and inhibitory neurons, in allowing GNE-8324 potentiation (see Fig.7b for a model). In addition, blockade of synaptic NMDARs by MK-801 in the absence of synaptic stimulation is also consistent with the notion that synaptic NMDARs in the inhibitory neurons have access to ambient glutamate while synaptic NMDARs on the excitatory neurons do not.

Based on these results and our prior study on GNE-8324, we propose the following model: (1) GNE-8324 binding is enhanced when NMDAR are in agonist-bound states. As the ambient synaptic glutamate level is very low in the excitatory neurons, virtually no NMDARs are bound by glutamate and hence no GNE-8324 binding. However, the higher ambient synaptic glutamate level in the inhibitory neurons allows GNE-8324 binding prior to synaptic transmission and readily potentiates NMDAR responses when synaptic transmission occurs. (2) The rate of GNE-8324 binding is slow evidenced by the slow on-rate kinetics^16^, relative to the very brief presence of glutamate in the synaptic cleft^61–63^. Therefore, GNE-8324 is normally ineffective at potentiating synaptic NMDARs on excitatory neurons, but can potentiate NMDARs on inhibitory neurons where ambient synaptic glutamate allows “pre-binding” before synaptic transmission.

There is a formal possibility that GNE-8324 can only potentiate synaptic NMDARs when glutamate concentration is low as an alternative explanation for our finding of puffed GNE-8324 potentiated NMDAR responses in excitatory neurons (Fig. 5d). Based on our experimental results, we suggest that this is not the case: (1) in the TBS experiments in the excitatory neurons (Fig. 5b) where the glutamate concentration is higher in the synaptic cleft than during low-frequency stimulation, we observed significant potentiation by GNE-8324. (2) In the d-APV dose–response experiments (Fig. 3a) where the apparent glutamate concentrations at synaptic NMDARs were reduced, we found an absence of GNE-8324 potentiation in the inhibitory neurons. (3) In our previous work, we reported that the efficacy of GNE-8324 is dependent on glutamate concentration in that higher glutamate enhances the potency of GNE-8324^16^. Based on these evidences from both our current results and the literature, we suggest the opposite that higher glutamate rather than lower glutamate enables GNE-8324 potentiation or increases its potentiation.

Our model is consistent with a compartmentalization between the ambient/extracellular and synaptic glutamate compartments, and this compartmentalization is more strongly present in the excitatory than in the inhibitory neurons. Consistent with this, Wu et al.^64^ found that in CA1 pyramidal neurons some extrasynaptic NMDARs on dendritic shafts, but not synaptic NMDARs, are bound by glutamate in the resting state and can be activated by back-propagating action potentials. This result suggests a lower ambient synaptic glutamate concentration compared to the ambient/extracellular level (outside synapses/spines). On the other hand, Chiu and Jahr^65^ found that glutamate concentrations at both the extracellular space and synaptic cleft in the medium spiny neurons of nucleus accumbens are in the tens nM range and there is unlikely to be a large gradient between these two compartments. While glutamate compartmentalization may differ between brain regions, the ambient/extracellular glutamate concentrations in acute brain slices (estimated to be between 25 and 90 nM^25,28,53^) is likely sufficient to facilitate GNE-8324 binding to NMDARs at rest, and a reduction in glutamate concentration at synaptic compartments could be sufficient to prevent potentiation.

In this study, we found substantial evidence for lower GLT-1 function at synapses onto inhibitory neurons. There is precedent for synapses onto different cell types being surrounded by different densities of GLT-1^39^. In addition, the wrapping of asymmetric (presumed glutamatergic) synapses by glial leaflets has been observed to be higher at dendritic spines compared to dendritic shafts in rat frontoparietal cortex^66^. This finding is consistent with greater glia wrapping and hence likely higher GLT-1 density surrounding spine synapses found on excitatory neurons vs. dendritic glutamatergic synapses on inhibitory neurons. Thus, the accessibility of ambient glutamate to synaptic NMDARs may be higher in inhibitory vs. excitatory neurons due to differences in glutamate transport, which is consistent with our data. In particular, we found evidence consistent with higher ambient glutamate level at synapses onto inhibitory neurons using both a glutamate-dependent NMDAR PAM and a use-dependent NMDAR blocker. This distinct role for ambient glutamate at synaptic NMDARs in inhibitory neurons may have important consequences for the different contributions of excitatory vs. inhibitory neurons during network function and information processing. More generally, our findings are consistent with a differential modulation of NMDARs by the distinct microenvironments surrounding glutamatergic synapses. For example, synaptic and extrasynaptic compartments have been shown to use distinct co-agonists for NMDAR activation, with d-serine for synaptic NMDARs and glycine for extrasynaptic NMDARs^67^. Our results using pharmacological probes and transporter manipulations indicate that heterogeneity of synaptic microenvironments is also contributed to by differences in neurotransmitter clearance. Future experiments using methods that could directly measure glutamate concentrations within the synaptic clefts of synapses onto different neuronal subtypes will be important for confirming and expanding this observation.

These findings provide insight into how to design allosteric modulators of synaptic glutamate receptors (AMPA, kainate, NMDA, and mGluR) that target inhibitory neurons over excitatory neurons in general: the ideal compound will have agonist-dependent association that is slow relative to the speed of synaptic activation of the receptors. We have observed other NMDAR PAMs that are much less agonist-dependent in their potency (e.g., GNE-6901 compared to GNE-8324^16^), and they potentiate NMDAR EPSCs in both excitatory and inhibitory neurons. Inhibitory neuron selectivity is unlikely to be absolute, but will depend on the degree of agonist dependence and the absolute potency of the modulator. For example, if solubility did not limit testing of GNE-8324, it is possible that very high concentrations would be sufficient to allow potentiation in excitatory neurons.

In this study, we gained insight into glutamate microenvironments of synapses by taking advantage of the glutamate dependence of GNE-8324. Other aspects of synaptic microenvironments could also vary between synapses, such as pH^68^, zinc concentration^69^, polyamine concentration, and so on, and therefore modulators with dependence on these aspects could also potentially exhibit synapse-specific effectiveness. For example, modulators with marked pH dependence, like UBP684^70^, or certain GluN2B antagonists^71^ could be used to probe for pH differences between synaptic microenvironments.

The above mechanism enables potentiation of NMDAR EPSCs by GNE-8324 in inhibitory neurons with low-frequency synaptic inputs. However, when inputs are of much higher frequencies (such as TBS), NMDAR EPSCs in the excitatory neurons can also be potentiated as we have shown here and this might represent an important mode of transmission in vivo. In addition, potentiation of extrasynaptic NMDARs by GNE-8324 on the excitatory neurons may modulate the excitability of these neurons^59,72,73^. Hence, in the context of in vivo brain function, compounds like GNE-8324 may exert effects via both synaptic and non-synaptic mechanisms in multiple cell types. While the overall effects on circuit function by compounds like GNE-8324 that preferentially enhance inhibitory vs. excitatory neuron activation in vivo remain to be tested (GNE-8324 has unfavorable pharmacokinetic properties), it is likely that such compounds will have distinct effects from NMDAR PAMs with equal ability to potentiate synaptic responses onto both neuronal types. PAMs with a GNE-8324-like mechanism may be particularly favorable for treating conditions involving inhibitory neuron hypofunction, as is thought to occur in schizophrenia and various other brain diseases^7–11^. Such an approach of enhancing excitatory synaptic drive to inhibitory neurons provides a mechanism for boosting inhibition that is distinct from enhancing the synaptic output of these neurons with drugs such as benzodiazepines.

In summary, the results of the present study using GNE-8324 as a tool reveal new aspects of glutamatergic synapse heterogeneity in the brain. It also reveals complexity of the function of NMDARs at synapses onto inhibitory vs. excitatory neurons and points to general concepts that could be exploited for developing therapeutics targeting inhibitory neurons.

## Methods

### Animals

Mice (C57BL/6J) were housed under standard conditions with free access to food and water. All experiments were carried out in accordance with the animal protection law and were approved by the Peking University Shenzhen Graduate School Animal Care and Use Committee. Mice of 6–10 weeks old, including wild-type (C57/BL-6) and GAD67-GFP transgenic mice, were anesthetized using phenobarbital sodium and decapitated. Mouse brains were quickly removed and placed in chilled ice-cold cutting solution (aCSF) containing (in mM): 110 choline chloride, 7 MgSO_4_, 2.5 KCl, 1.25 NaH_2_PO_4_,25 NaHCO_3_, 25 d-glucose, 11.6 sodium ascorbate, 3.1 sodium pyruvate, and 0.5 CaCl_2_ gassed with 95% O_2_ and 5% CO_2_. Coronal frontal sections (400 μm) were cut on a DTK-1000 tissue slicer (DTK, Japan) in 4 °C cutting aCSF. Slices were allowed to recover for 30 min at 32 °C, and then transferred to a holding chamber at room temperature in aCSF containing (in mM): 127 NaCl, 2.5 KCl, 1.25 NaH_2_PO_4_, 25 NaHCO_3_, 25 d-glucose, 2 CaCl_2_, and 1 MgSO_4_. Recording started at least 1 h after recovery. All animals were randomly used for all experiments.

### Electrophysiological recordings and analysis in brain slices

Individual slices were transferred to the recording chamber on an Olympus microscope (BX51WI) with a ×40 water-immersion differential interference contrast objective. Slices were constantly perfused at either room temperature (23–26 °C) or near physiological temperature (32 ± 1 °C or 35 ± 1 °C) with oxygenated aCSF (4–5 ml/min). Recordings were made from PFC neurons which were electrically stimulated every 30 s using glass pipette positioned about 50–80 μm from the recorded neurons (World precision Instruments, 1–2 MΩ, filled with aCSF). Recording pipettes (4–8 MΩ) were filled with (in mM): 125 CsMeSO_4_, 5 NaCl, 1.1 EGTA, 10 HEPES, 0.3 Na_2_GTP, 4 Mg-ATP, and 5 QX-314. Recordings were made from layer 2/3 of the PFC, in a depth of about 50–100 μm in the slices, not different between excitatory and inhibitory neurons. GABAergic inhibitory neurons were identified using GFP fluorescence in GAD67-GFP transgenic mice, with no distinction between different subtypes of inhibitory neurons. Pyramidal neurons were identified by their apical dendrite and triangular somata. Data were acquired using HEKA EPC10 double patch clamp amplifier (HEKA). Signals were acquired at a sampling rate of 10 kHz and filtered at 2 kHz. Series resistance of the recording pipette was between 10 and 25 MΩ, and monitored throughout the experiments. Experiments were discarded if changes in series resistance exceeded 30%. Neurons with holding current >−200 pA (at −60 mV) were excluded from the data analysis. Experiments were not performed in a double-blind manner.

To isolate GluN2A NMDAR EPSCs, neurons were held at −60 mV in the presence of NBQX (10 μM), picrotoxin (50 μM), Mg^2+^ (0.5 mM), and piperindine 18 (1 μM; selective GluN2B-NMDAR antagonist). All drugs were applied in the bath. For experiments testing the effect of CPG on holding current and MK-801 on ambient glutamate responses, neurons were held at +40 mV. After obtaining whole-cell configuration, 5–10 min was allowed for recordings to stabilize. In experiments using γ-DGG, 10 or 100 μM d-APV was used to block NMDAR. TBS (five pulses at 250 Hz) was delivered every 90 s to avoid inducing synaptic plasticity. For puffing experiments, glass pipettes (1–2 MΩ) filled with d-APV (5 mM) or GNE-8324 (3 mM) were positioned ~50 μm from the recorded neuron, with pressure (10 psi for 50 ms) from a Picospritzer III (Parker). For measuring the ambient NMDAR responses with CPG incubation (50 μM, >20 min), d-APV was fast perfused into the recording chamber by a tube positioned at the edge of the recording chamber. In the experiments where NMDA was bath applied, both 5 and 7 μM NMDA was used either on the same neurons or on different neurons (each neuron was exposed to either 5 or 7 μM NMDA, but not both). Similar changes in the holding current and noise were seen between these two conditions, and hence results were pooled. For testing the effect of bath-applied NMDA on GNE-8324, neurons were exposed to either 5 or 7 μM NMDA, but not both. In the DHK at 32 °C experiment, 150 μM DHK was used to maintain an adequate recording conditions (such as voltage control).

NMDAR EPSCs were analyzed off-line using Clampfit software (MDS Analytical Technologies). Average of NMDAR EPSCs in 2–5-ms window centered on the maximal response region was taken as peak amplitude. For measuring area, EPSC was integrated from its start to when it decays to baseline level. Stimulus artifacts were not subtracted in these measurements. For measuring NMDAR EPSC kinetics, rise time was taken as time taken from 10 to 90% of maximal responses, and decay time (*τ*) was fitted by a single exponential function. For the d-APV dose–responses experiments, the non-d-APV-sensitive components (mostly stimulus artifacts) were subtracted from the traces before quantification. Dose–responses of d-APV were fitted with the Hill equation. For measuring noise and holding currents in the 5 or 7 μM NMDA experiments, noise was quantified by calculating the standard deviation of baseline current, and holding current was quantified by calculating the absolute mean value. Changes in noise and/or holding current were quantified by comparing the values before and after bath application of NMDA, d-APV, or GNE-8324 (puffed).

Statistical significance was assessed using an unpaired *t* test or ANOVA followed by Dunnett’s or Tukey's tests, and paired *t* test was used for puffing GNE-8324 experiment. Normalized values were calculated as percentage change over the baseline. Data values are presented as mean ± SEM. Statistical significance was established as *P* *<* 0.05 (*), *P* *<* 0.01 (**), and *P* *<* 0.001 (***).

### Electrophysiological recordings of GluN1/2A/2D* NMDARs

Triheteromeric GluN1/2A/2D* NMDARs were expressed and functionally isolated as described^58^. In brief, *Xenopus laevis* oocytes were injected with a mixture of GluN1, GluN2A, and GluN2D* subunit, with GluN2D* indicating the GluN2D subunit carrying the N639K mutation which suppresses block by extracellular Mg^2+^. This results in the assembly and expression of three distinct receptor population at the cell surface: GluN1/2A and GluN1/2D* diheteromers, and GluN1/2A/2D* triheteromers. Because of their greatly reduced single-channel conductance, GluN1/2D* diheteromers contribute minimally to the total NMDAR responses (likely <1% of the total NMDAR current; in parallel experiments, we found that currents from GluN1/2D*-expressing oocytes were on average 300-fold small than those from GluN1/2A-expressing oocytes, *N* = 20–24). Diheteromeric GluN1/2A NMDARs can be fully inhibited by adding 1 mM Mg^2+^ into the extracellular recording solution and setting the holding potential to −80 mV. Accordingly, under these conditions, the remaining Mg^2+^-insensitive current (about 20% of the initial current recorded in the absence of Mg^2+^) is almost entirely, if not entirely, carried by triheteromeric GluN1/2A/2D* NMDARs (which display reduced Mg^2+^sensitivity compared to wild-type GluN1/2A or GluN1/2D NMDARs).

The rat GluN1, GluN2A, and GluN2D subunits were used. Oocytes were prepared and injected with cRNAs obtained using mMessage/mMachine (Ambion), and currents measured 2–4 days later using two-electrode voltage clamp, as previously described^74^. The external solution contained (in mM): 100 NaCl, 2.5 KCl, 0.3 BaCl_2_, 5 HEPES, and 1 MgCl_2_. For GNE-8324 dose–response experiments, recordings were performed in the presence of 100 µM glycine and 0.3 µM glutamate. Glutamate dose–response recordings were performed in the presence of 100 µM glycine. Experiments were performed at −30 mV for oocytes expressing diheteromeric GluN1/2A NMDARs, and at −80 mV for oocytes expressing either diheteromeric GluN1/2D* NMDARs or triheteromeric GluN1/2A/2D* NMDARs. NAB-14 experiments were performed in the presence of 100 µM glycine and 100 µM glutamate, and at a holding potential of −30 mV (GluN1/2A NMDARs) and −80 mV (GluN1/2D* and GluN1/2A/2D* NMDARs). All data are mean ± SEM.

### Western blot

After 5 consecutive days of injection of Cef in male C57/BL-6 mice, mice were anesthetized and perfused with phosphate-buffered saline (PBS) through the heart. Freshly dissected mouse brains were treated with RIPA lysis and homogenized by S10- High Speed Homogenizer (Xinzhi Biotechnology Co., Ltd., China), and then centrifuged at 17,925 × *g* for 10 min at 4 °C. Protein concentration was measured with a BCA Protein Assay Kit (Pierce). Clarified cell extracts were mixed with 6× sodium dodecyl sulfate (SDS) sample buffer. Protein samples were run on 8% SDS-polyacrylamide gel electrophoresis using a Bio-Rad gel system and transferred onto nitrocellulose membranes. Loading controls (glyceraldehyde 3-phosphate dehydrogenase (GAPDH)) were run on the same gel. Membranes were then probed with antibodies with the appropriate dilutions, including anti-GLT-1 (1:2000; Abcam, catalog no. ab178401) and anti-GADPH (1:10,000; Sigma, catalog no. G8795). ImageJ was used for densitometric analysis. Experiments were performed in a double-blind manner.

### Materials/drugs

Materials/dugs used in this study have been obtained as: QX-314 chloride, d-APV, γ-DGG, NBQX, (*S*)-4-CPG, DHK, and dl-TBOA were from Tocris; NMDA, MK-801, and picrotoxin were from Sigma-Aldrich; Cef was from USP; NAB-14 from two sources was used: a generous gift from Dr. Stephen Traynelis (Emory University, Atlanta, GA, USA) and own synthesis at PKUSZ. GNE-8324 was provided by Genentech (South San Francisco, CA, USA).

## Electronic supplementary material

Supplementary Information

## Data Availability

All relevant data are available from the authors.
