# Author Correction: Higher ambient synaptic glutamate at inhibitory versus excitatory neurons differentially impacts NMDA receptor activity

**DOI:** 10.1038/s41467-018-07441-1

**Published:** 2018-11-15

**Authors:** Lulu Yao, Teddy Grand, Jesse E. Hanson, Pierre Paoletti, Qiang Zhou

**Affiliations:** 1 School of Chemical Biology and Biotechnology, Peking University Shenzhen Graduate School, 518055 Shenzhen, China; 2 Institut de Biologie de l’Ecole Normale Supérieure (IBENS), Ecole Normale Supérieure, CNRS, INSERM, Université PSL, 46 rue d’Ulm, 75005 Paris, France; 3 Department of Neuroscience, Genentech, Inc., South San Francisco, 94080 CA USA

Correction to: *Nature Communications*; 10.1038/s41467-018-06512-7, published online 01 October 2018

The original version of this Article omitted the middle initial of the author Jesse E. Hanson. This has now been corrected in both the PDF and HTML versions of the Article.

