# Supplementary material for: Higher ambient synaptic glutamate at inhibitory versus excitatory neurons differentially impacts NMDA receptor activity
Source: Nat Commun. 2018 Oct 1;9:4000. doi: 10.1038/s41467-018-06512-7 (PMC6167324; doi:10.1038/s41467-018-06512-7)
Supplement: Supplementary file 1 — Supplementary Information [file 41467_2018_6512_MOESM1_ESM.pdf]

Higher ambient synaptic glutamate at inhibitory versus excitatory neurons differentially impacts NMDA receptor activity

Yao et al.

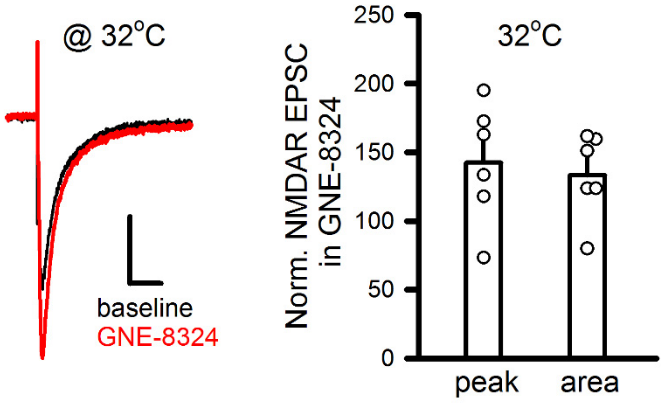

Supplementary Figure 1. Potentiation of NMDAR EPSCs by GNE-8324 at 32 °C.

N=6, 142.6±17.86% (peak), 133.3±12.7% (area). Error bars represent SEM.

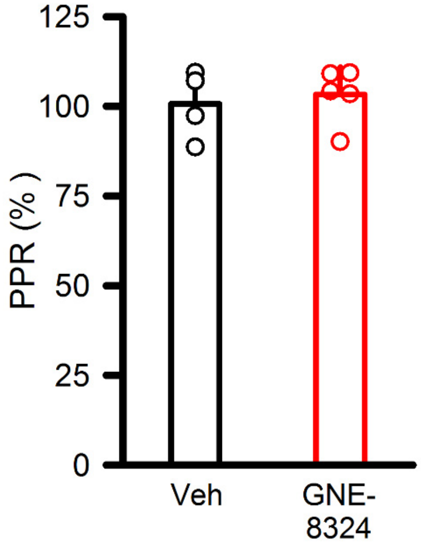

Supplementary Figure 2. No effect of GNE-8324 on the paired-pulse ratio (PPR) of AMPAR-EPSCs.

N=4, 5, for Veh and GNE-8324, 100.6±4.77% (Veh), 103.2±3.49% (GNE-8324). P=0.67, unpaired t-test. Error bars represent SEM.

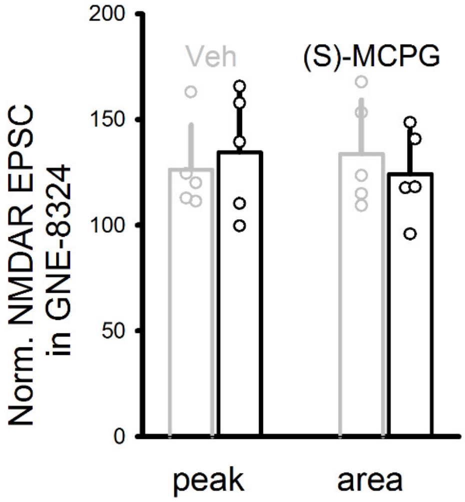

Supplementary Figure 3. GNE-8324 potentiation of NMDAR EPSCs in inhibitory neurons was not affected by (S)-MCPG.

N=5, 5, for (S)-MCPG and Veh. For peak, 126.3±9.48% (Veh), 134.5±12.91% ((S)-MCPG); for area, 133.7±11.38% (Veh), 124.1±9.34% ((S)-MCPG). P (peak) =0.62, P (area) =0.53, unpaired t-test. Error bars represent SEM.

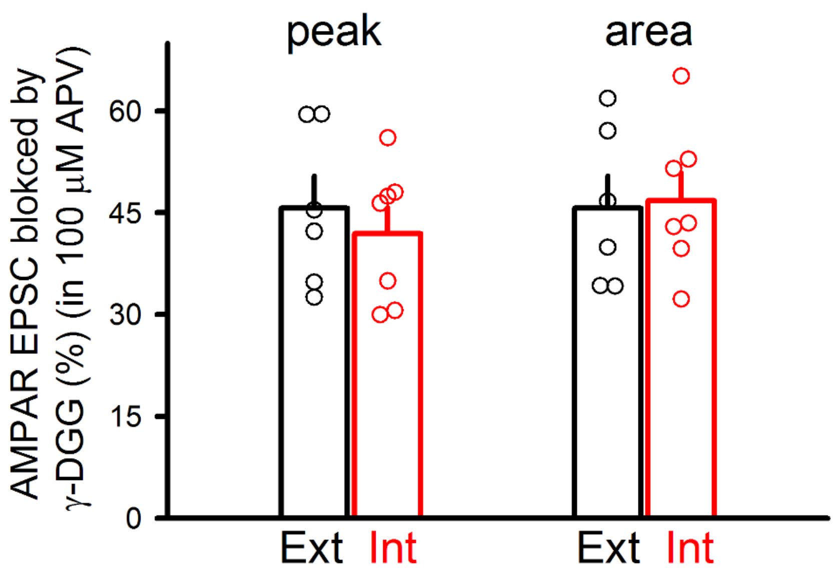

Supplementary Figure 4. Effects of γ-DGG (0.4 mM) on AMPAR EPSCs in excitatory and inhibitory neurons in the presence of high concentration of D-APV.

No difference was found between either peak or area of AMPAR EPSCs in 100 μM D-APV. N = 6, 7 for excitatory and inhibitory neurons. For peak, 45.66±4.78% (Ext), 41.92±3.80% (Int); for area, 45.66±4.78% (Ext), 46.84±4.03 (Int). P (peak) =0.54, P (area) =0.85, unpaired t-test. Error bars represent SEM.

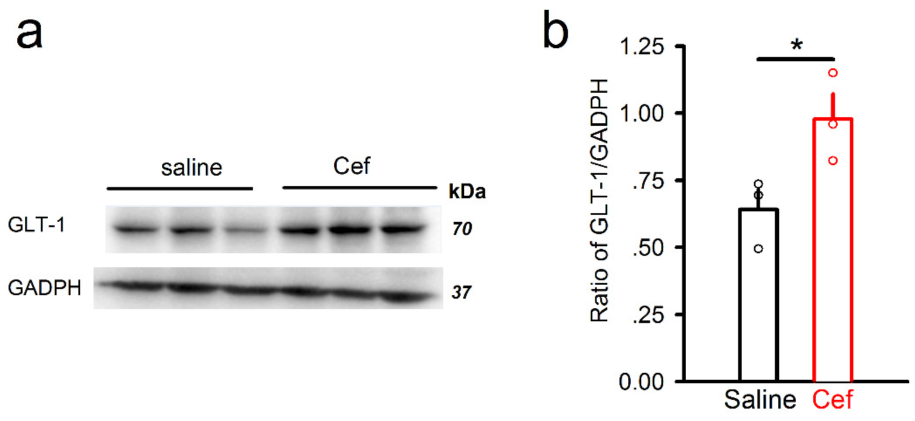

Supplementary Figure 5. Injection of ceftriaxone led to elevated expression of GLT-1.

a. Sample Western Blot image of GLT-1 levels in mice injected with either ceftriaxone (Cef) or vehicle for 5 consecutive days (Molecular weight, GLT-1, 70k Da; GADPH, 37k Da).

b. Quantification of the WB results (N = 3 mice each), 0.64±0.074 (Saline), 0.98±0.095 (Cef). *, P < 0.05, unpaired t-test. Error bars represent SEM.

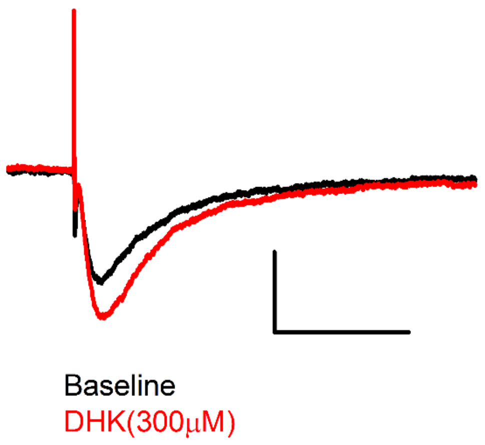

Supplementary Figure 6. DHK affects NMDAR EPSCs.

Sample traces of NMDAR EPSCs before and after application of DHK in an excitatory neuron.

Scale bars, 50 pA/100 ms.

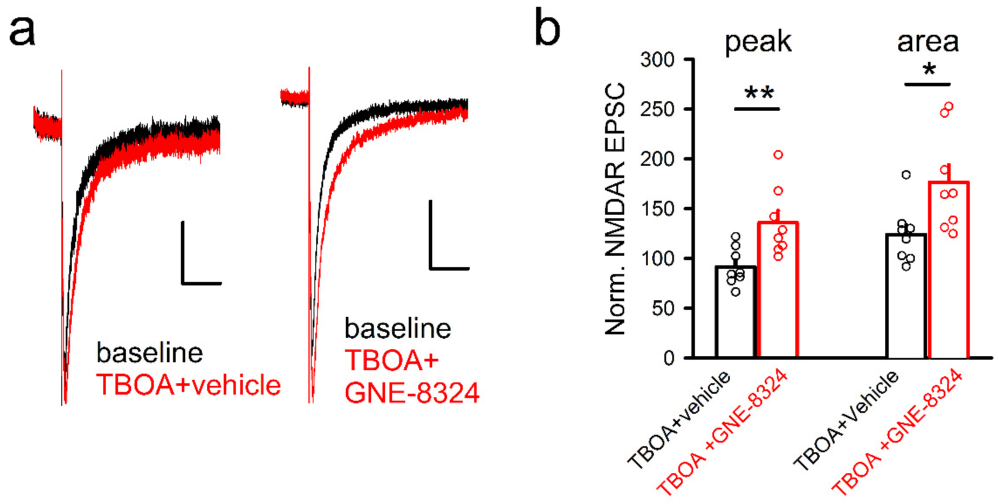

Supplementary Figure 7. TBOA enabled GNE-8324 potentiation of NMDAR EPSCs in excitatory neurons.

a. Sample traces of NMDAR EPSCs in response to bath application of either TBOA (10 μM) or TBOA+GNE8324. Scale bars, 10 pA/200 msec (left); 20 pA/200 msec (right).

b. Significant potentiation of GNE-8324 on NMDAR EPSCs in the presence of TBOA. P = 8, 8 for TBOA and TBOA+GNE-8324. For peak, 91.46±6.70% (TBOA+vehicle), 135.8±12.25% (TBOA+GNE-8324); for area, 123.9±10.24% (TBOA+vehicle), 176.5±17.55 (TBOA+GNE-8324). **, P < 0.01; *, P < 0.05, unpaired t-test. Error bars represent SEM.

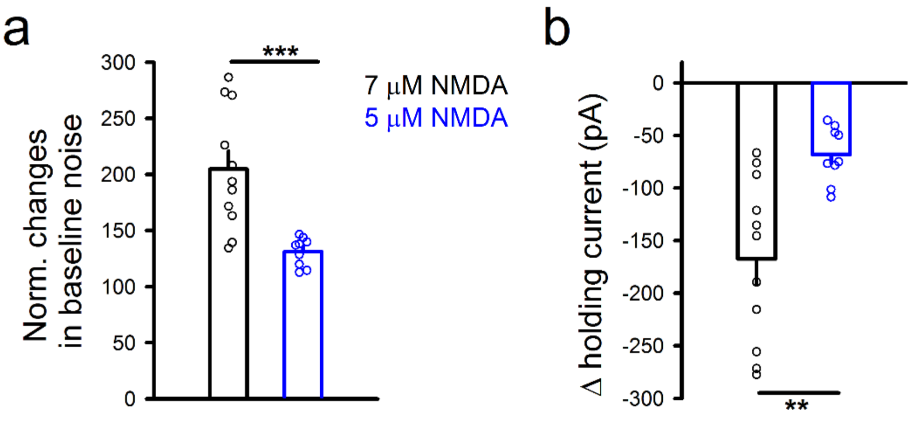

Supplementary Figure 8. Exogenous NMDA application resulted in an increase in baseline noise and holding current.

Quantification of the baseline noise (a) and holding current (b) in the presence of 5 μM or 7 μM NMDA. Both were normalized to baseline prior to NMDA application. N = 9, 11 for 5 μM and 7 μM NMDA. For normalized changes in baseline noise, 204.8±16.17% (7μM NMDA), 131.2±4.25% (5μM NMDA); for Δholding current, -167.4±23.77pA (7μM NMDA), 68.1±8.80pA (5μM NMDA). ***, $P < 0.001$; **, $P < 0.01$, unpaired t-test. Error bars represent SEM.

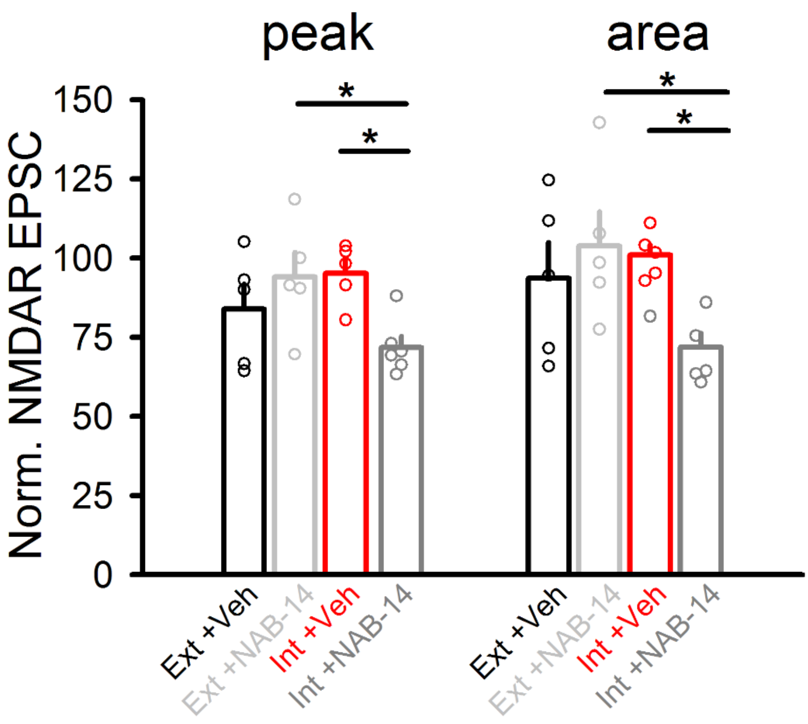

Supplementary Figure 9. Effect of NAB-14 on NMDAR EPSCs in excitatory and inhibitory neurons.

NAB-14 or vehicle was bath applied onto either excitatory or inhibitory neurons. NMDAR EPSCs were normalized to the pre-drug baseline level. N = 5,5,5,6 for Ext/Veh, Ext/NAB-14, Int/Veh, Int/NAB-14. $P < 0.05$, one-way ANOVA with Dunnett correction. Error bars represent SEM.

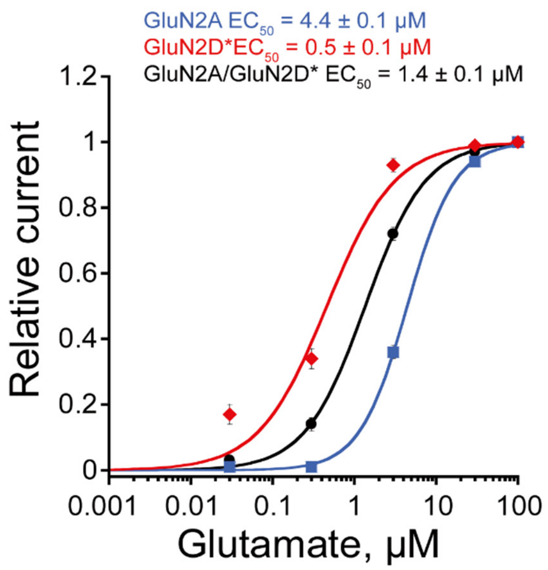

Supplementary Figure 10. Glutamate dose-response curves for diheteromeric GluN1/2A NMDARs, diheteromeric GluN1/2D* NMDARs or triheteromeric GluN1/2A/2D* NMDARs. Glutamate EC50 and Hill coefficient (nH) values are, respectively: 4.4 ± 0.1 µM and 1.5 (N=4); 0.5 ± 0.1 µM and 1.0 (N=3); and 1.4 ± 0.1 µM and 1.2 (N=7). Error bars represent SEM.
